# Supplementary figures and images for: Sequential Treatment with Temozolomide Plus Naturally Derived AT101 as an Alternative Therapeutic Strategy: Insights into Chemoresistance Mechanisms of Surviving Glioblastoma Cells
Source: Int J Mol Sci. 2023 May 22;24(10):9075. doi: 10.3390/ijms24109075 (PMC10218802; doi:10.3390/ijms24109075)

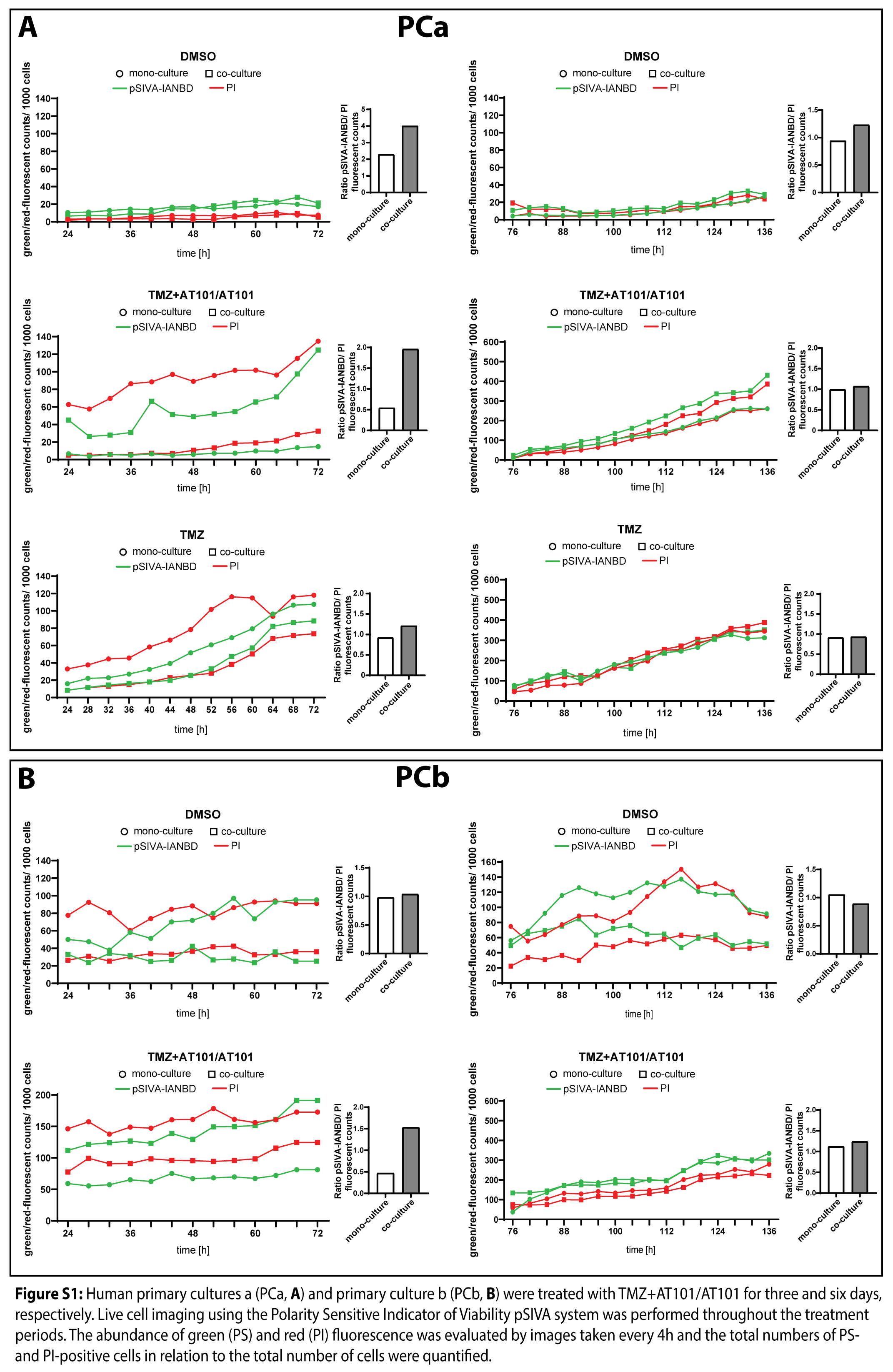

Supplement: Supplementary file 1 [file ijms-24-09075-s001.zip › FigureS1.tif]

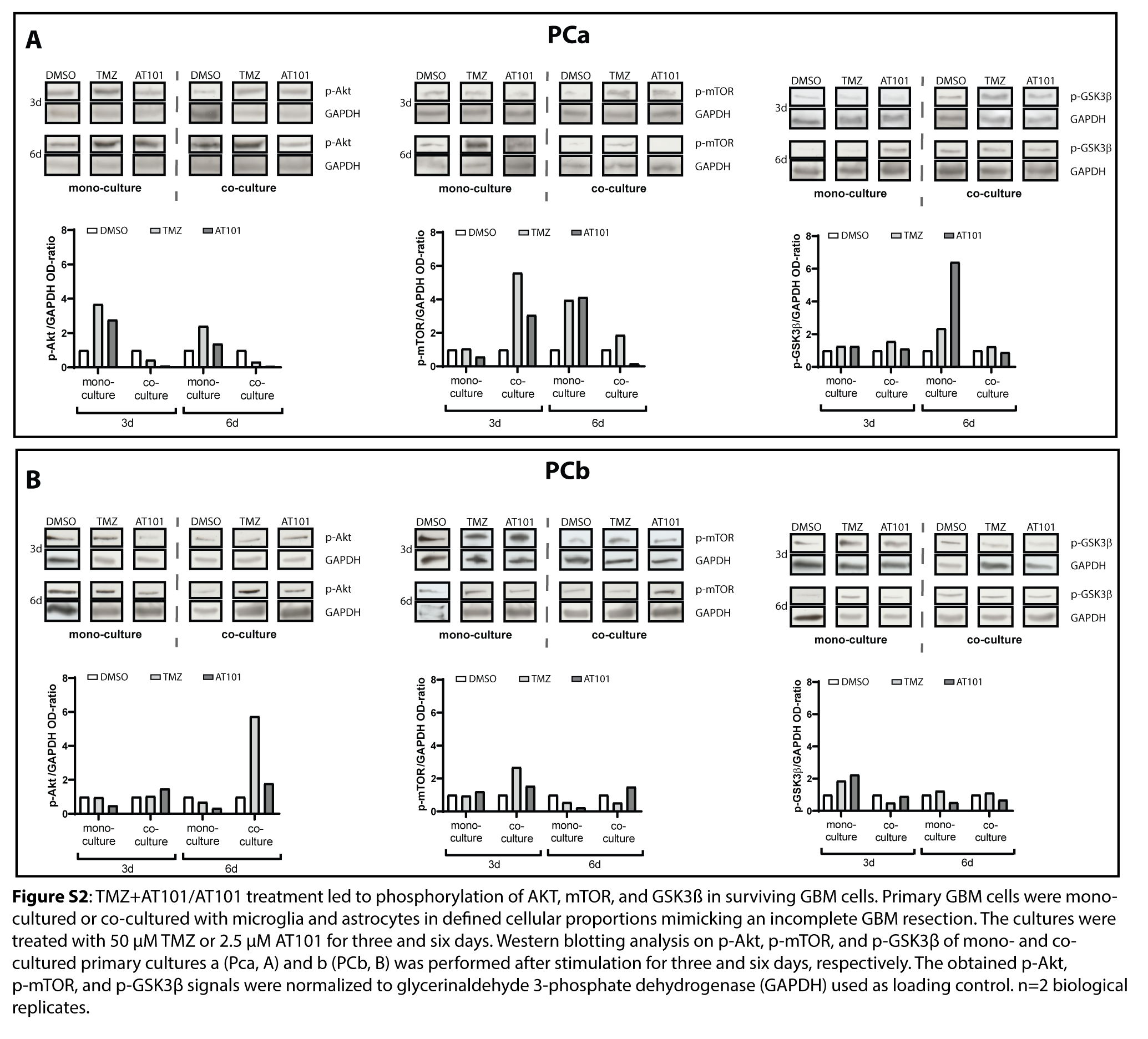

Supplement: Supplementary file 1 [file ijms-24-09075-s001.zip › FigureS2.tif]

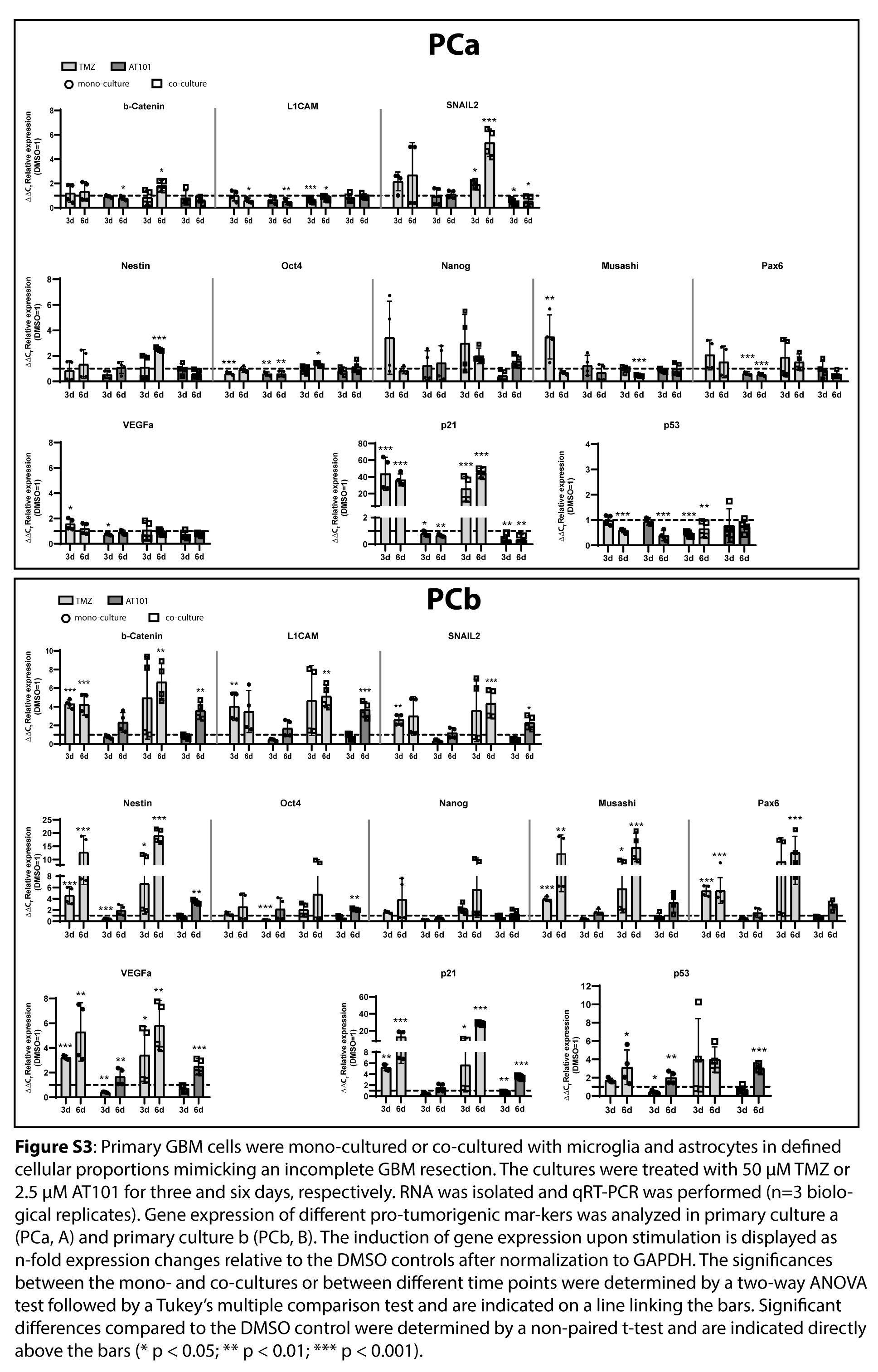

Supplement: Supplementary file 1 [file ijms-24-09075-s001.zip › FigureS3.tif]

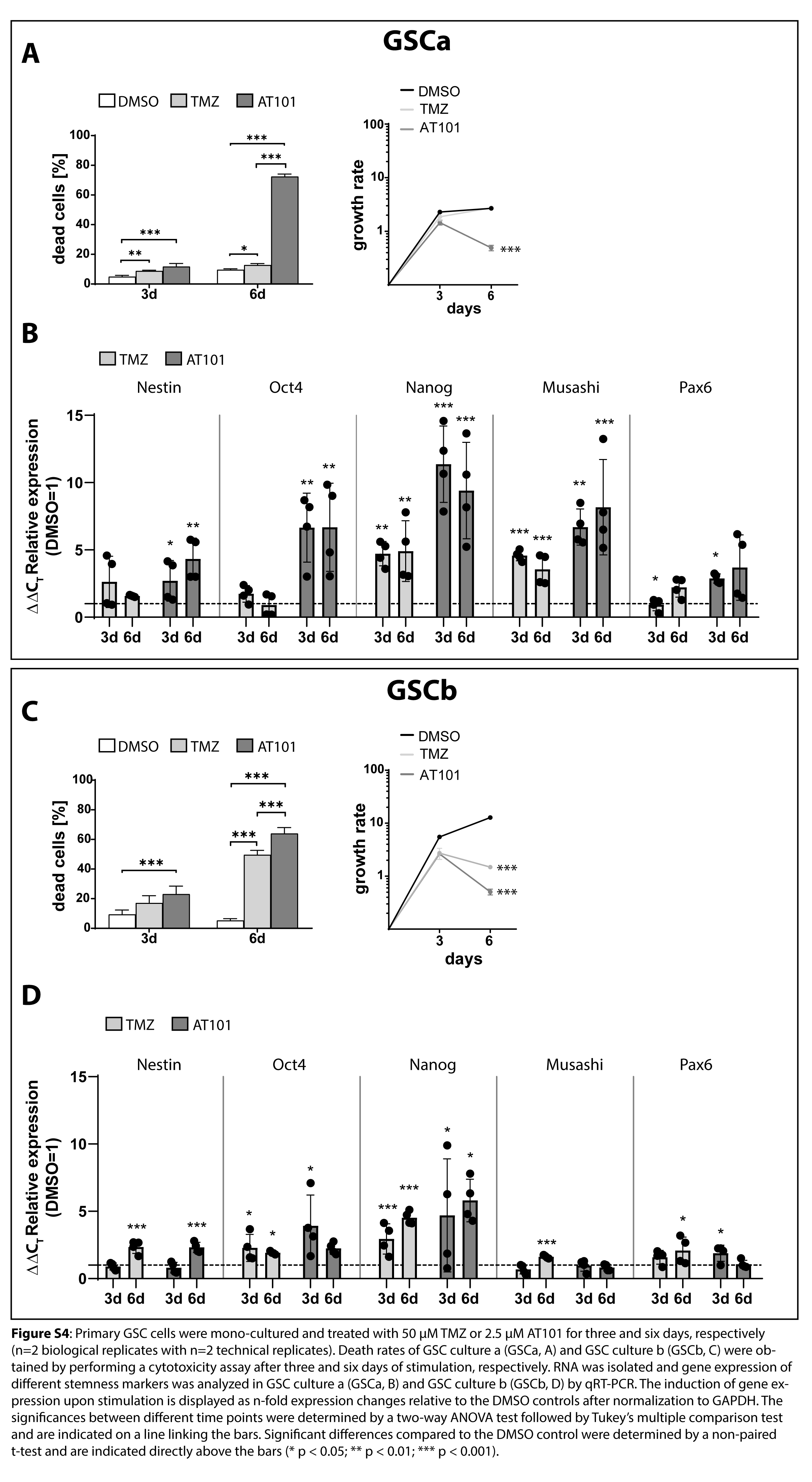

Supplement: Supplementary file 1 [file ijms-24-09075-s001.zip › FigureS4.tif]

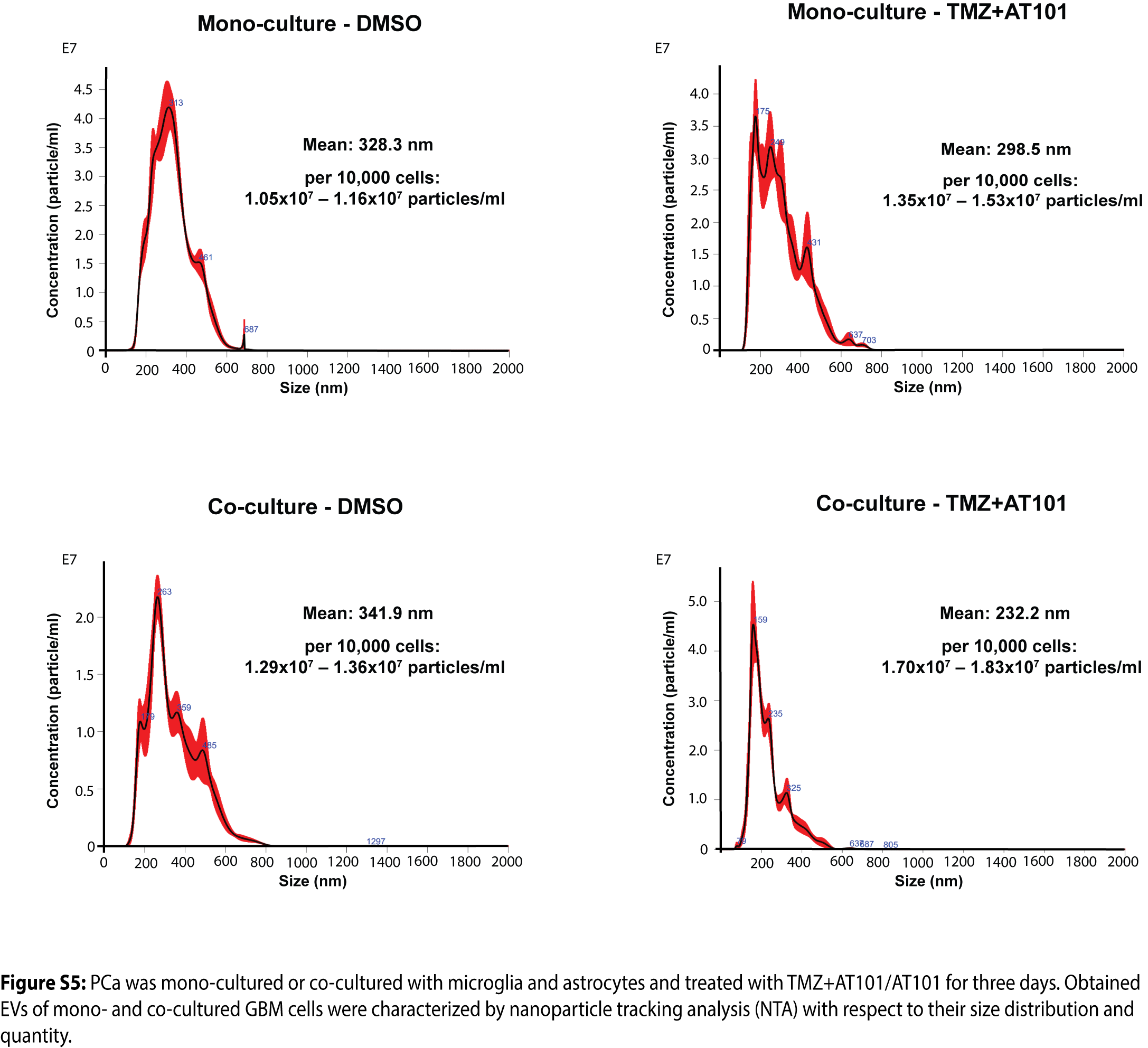

Supplement: Supplementary file 1 [file ijms-24-09075-s001.zip › FigureS5.tif]
